# Clinical implications of aminotransferase elevation in hospitalised infants aged 8‐90 days with respiratory virus detection

**DOI:** 10.1111/irv.12732

**Published:** 2020-03-10

**Authors:** Sang Gyeom Kim, Yu Na Oh, Joon Kee Lee

**Affiliations:** ^1^ Department of Pediatrics Chungbuk National University Hospital Cheongju South Korea

**Keywords:** aminotransferase, hepatitis, respiratory tract infections

## Abstract

**Background:**

Fever and respiratory symptoms are the major causes of hospitalisation in infants aged 90 days or less. Respiratory viruses (RVs) are detected by multiplex reverse transcriptase‐polymerase chain reaction (mRT‐PCR) in up to 70% of infants tested in this population. Aminotransferase elevation is not uncommon in RV infections, and repeat laboratory investigations are frequent due to concerns regarding the occurrence of hepatic disease.

**Methods:**

This retrospective observational cohort study included 271 infants aged 8‐90 days, with positive RV mRT‐PCR results. Data were obtained on demographics, laboratory results and final diagnoses of hepatobiliary disease.

**Results:**

Fever (73.1%) and/or respiratory symptoms (75.6%) were the major presentations among the hospitalised infants. Aspartate aminotransferase (AST) or alanine aminotransferase (ALT) levels were elevated in 62 (22.9%) of the 271 infants. Twenty‐four of these 62 infants had their first follow‐up, and 19 (79.2%) showed persistent elevation. All 10 (100%) infants who had their second follow‐up showed persistently elevated aminotransferase levels. Eventually, none of the 10 infants were diagnosed with hepatic disease during the median follow‐up of 10 days (range 3‐232 days). Among the RVs of interest, parainfluenza virus type 1 was significantly associated with aminotransferase elevation (odds ratio: 2.95; 95% confidence interval [CI]: 1.11‐7.83).

**Conclusions:**

RV‐related non‐specific hepatitis is occasionally observed in infants aged 8‐90 days, and ALT elevation is the most common abnormality. However, a final diagnosis of primary hepatobiliary disease appears to be rare. Therefore, regular follow‐ups and targeted testing may be recommended in this specific population.

## 1 INTRODUCTION

Respiratory viruses (RVs) involved in childhood lower respiratory tract infections can be identified using the multiplex reverse transcriptase‐polymerase chain reaction (mRT‐PCR).^1^, ^2^ Despite the lack of clear guidelines for the use of mRT‐PCR in the management of febrile infants aged younger than 90 days, this diagnostic modality is frequently used to reduce antibiotic use, length of hospital stay and laboratory investigations and for aiding decision‐making by doctors and families in certain cases.^3^, ^4^, ^5^, ^6^

The guidelines for the management of febrile infants aged 90 days or less vary from one institution to another. In general, the mainstay of management involves hospital admission and the administration of empiric antibiotics.^7^ The hospital admission panel or battery measures the basic laboratory parameters of a patient at admission, that commonly includes aminotransferase measurements.^8^ RV infections are known causes of non‐specific hepatitis, which present with aminotransferase elevation.^9^ Non‐specific hepatitis is not uncommon even with a wide range of “normal” aminotransferase levels in this specific age group. Owing to the diagnostic possibility of true hepatic disease, additional laboratory follow‐ups are performed based on the care providers’ preference. Such investigations may prolong the duration of hospital stay and impart undue stress to both, the baby and the caregiver.^10^

The main objective of the study was to evaluate the true clinical implications of aminotransferase elevation in infants aged 8‐90 days hospitalised with RV infections. The association between the type of RV and aminotransferase elevation was also investigated.

## 2 METHODS

### 2.1 Patient characteristics

This retrospective observational cohort study included infants aged 8‐90 days with positive RV mRT‐PCR results, who were admitted to the Department of Pediatrics at the Chungbuk National University Hospital between January 2014 and December 2018. Infants born in the hospital and those with positive results for multiple RVs were excluded. Infants aged less than 8 days were excluded based on the incubation period of the viruses. The upper limit (90 days) was set based on the well‐recognized fact that febrile infants aged between 0 and 90 days are at a higher risk of severe bacterial infections (SBIs) than infants and children aged between 3 and 36 months.^11^

Basic demographic data, laboratory and imaging results, data on the final diagnosis of hepatic disease, if any, and mortality data were collected. Information was also obtained regarding the initial presentations of the infants. Fever was defined as a tympanic temperature of at least 38.0°C, as measured by medical personnel in the institute. Perceptions of fever by the caregiver or temperatures measured outside the hospital were excluded. Complete blood cell counts, aminotransferase levels, bilirubin, albumin and the coagulation panel were tested. For infants with elevated aminotransferases, data on the subsequent follow‐up and laboratory tests were collected. Aminotransferase levels of infants with aminotransferase elevation at each follow‐up stage were compared. Aminotransferase levels were also compared between infants who did and did not require follow‐up.

### 2.2 Criteria for elevated aminotransferase levels

The normal ranges of aspartate aminotransferase (AST) and alanine aminotransferase (ALT) varied with sex and age, and were defined as follows: for AST, less than 72 IU/L for infants younger than 1 month and less than 64 IU/L for infants older than 1 month, and for ALT, less than 41 IU/L for boys younger than 1 month, less than 33 IU/L for girls younger than 1 month and less than 46 IU/L for both, boys and girls older than 1 month.^12^ The aminotransferase elevations in patients were defined as elevations of either AST or ALT, unless otherwise specified.

### 2.3 Respiratory virus identification

The presence of fever and/or respiratory symptom(s) was the major indication of a RV, which was subsequently identified using mRT‐PCR. The decision to perform the test was made independently by the attending physician. Nasopharyngeal swab specimens were collected. The PCR assay kit (Allplex™ Respiratory Panel Assays, Seegene Inc) was used for virologic diagnosis of adenovirus (AdV), rhinovirus (HRV), enterovirus (HEV), influenza A virus (FluA), influenza B virus (FluB), parainfluenza virus 1 (PIV1), parainfluenza virus 2 (PIV2), parainfluenza virus 3 (PIV3), parainfluenza virus 4 (PIV4), respiratory syncytial virus A (RSV A), respiratory syncytial virus B (RSV B), bocavirus 1/2/3/4 (HBoV), metapneumovirus (MPV), coronavirus 229E (CoV‐229E), coronavirus NL63 (CoV‐NL63) and coronavirus OC43 (CoV‐OC43).

### 2.4 Statistical analyses and sample size calculations

Statistical analyses were performed using SPSS Statistics for Windows version 25.0 (IBM Corp.). Demographic, clinical and laboratory data were evaluated and presented descriptively. Continuous data were analysed using the paired sample t test or the Mann‐Whitney *U* test. Categorical data were analysed using the chi‐squared test or Fisher's exact test. *P *< .05 was considered statistically significant.

Minimum sample sizes for detecting mean differences with sufficient statistical power (*α = *0.05, *β = *80%) were calculated and assured at each stage of the laboratory test in groups with and without aminotransferase elevation. For viruses associated with significant aminotransferase elevation, statistical power for the sample sizes was calculated separately.

### 2.5 Ethical consideration

The institutional review board of the Chungbuk National University Hospital approved the study protocol (IRB no.2019‐01‐002). The need for informed consent was waived due to the retrospective nature of the study and data anonymisation.

## 3 RESULTS

### 3.1 Patient characteristics

Among the 271 infants included in the study between January 2014 and December 2018, 159 (58.7%) were male and 112 (41.3%) were female, with a mean age of 54.2 ± 21.7 days (Table 1).

**Table 1:** Demographic and clinical characteristics of infants with normal and elevated aminotransferases

Characteristics	Total (N = 271)	Normal aminotransferase (n = 209)	Elevated aminotransferase (n = 62)	*P* value
Demographics				
Age (days)	54.2 ± 21.7	52.1 ± 21.8	61.5 ± 19.7	.**003**
Male, No. (%)	159 (58.7%)	127 (60.8%)	32 (51.6%)	.199
Hospital stay (days)	6.1 ± 2.3	6.0 ± 1.8	6.1 ± 2.4	.716
Febrile contact, No. (%)	140 (51.7%)	109 (52.2%)	31 (50.0%)	.773
Laboratory				
Complete blood count				
White blood cell (cells/µL)	11 235.6 ± 4678.4	10 837.2 ± 4535.3	12 565.6 ± 4935.7	**.010**
Neutrophil (%)	34.5 ± 16.3	34.7 ± 16.5	33.8 ± 15.6	.708
Lymphocyte (%)	48.8 ± 15.9	48.2 ± 15.7	50.7 ± 16.5	.308
Haemoglobin (g/dL)	11.3 ± 1.5	11.2 ± 1.5	11.4 ± 1.6	.326
Platelet (×10^3^/µL)	393.1 ± 137.3	389.1 ± 138.3	406.6 ± 134.2	.380
Chemistry				
AST (IU/L)	55.8 ± 111.7	34.1 ± 9.5	129.0 ± 218.7	**.001**
ALT (IU/L)	48.0 ± 103.1	23.2 ± 7.9	131.4 ± 194.0	**<.001**
Albumin (g/dL)	4.2 ± 0.3	4.2 ± 0.3	4.3 ± 0.2	**.029**
Total bilirubin (mg/dL)	1.8 ± 2.6	1.9 ± 2.5	1.5 ± 2.7	.273
C‐reactive protein (mg/dL)	0.9 ± 1.3	0.9 ± 1.4	0.7 ± 0.9	.094
Diagnosis				
LRTI	86 (31.7%)	67 (32.1%)	19 (31.7%)	.878
Urinary Tract Infection	14 (5.2%)	12 (5.7%)	2 (3.2%)	.744
Meningitis	9 (3.3%)	8 (3.8%)	1 (1.6%)	.689

Note

Values are presented as mean ± standard deviation, unless specified otherwise.

Abbreviations: AST, aspartate aminotransferase; ALT, alanine aminotransferase; LRTI, lower respiratory tract infection. P < .05 are marked in bold.

*P* < .05 are marked in bold.

Fever (198 of 271, 73.1%) and/or respiratory symptoms (205 of 271, 75.6%) were the major presentations among the hospitalised infants. Fever without any associated symptoms was observed in 43 (15.9%) infants at presentation. Only two infants (0.7%) had no fever or respiratory symptoms and presented with seizure and rash; 140 (51.7%) infants had a history of contact with a febrile person. The mean duration of hospital stay was 6.1 ± 2.3 days. Following upper respiratory tract infection, lower respiratory tract infection was the most common diagnosis (31.7%) followed by urinary tract infection (5.2%) and meningitis (3.3%).

The viruses detected using mRT‐PCR included the following: AdV, 9 (3.3%); HRV, 46 (17.0%); HEV, 35 (12.9%); FluA, 32 (11.8%); FluB, 3 (1.1%); PIV1, 18 (6.6%); PIV2, 3 (1.1%); PIV3, 19 (7.0%); PIV4, 12 (4.4%); RSV A, 38 (14.0%); RSV B, 10 (3.7%); HBoV, 8 (3.0%); MPV, 11 (4.1%); CoV‐229E, 1 (0.4%); CoV‐NL63, 8 (3.0%); and CoV‐OC43, 18 (6.6%).

### 3.2 Initial laboratory results

At admission, the mean AST was 55.8 ± 111.7 IU/L and ALT was 48.0 ± 103.1 IU/L. Compared to age‐based reference values, AST and ALT elevations were present in 36 (13.3%) and 60 (22.1%) of the 271 infants, respectively. Overall, 34 of the 271 (12.5%) infants showed an increase in both AST and ALT levels and 62 (22.9%) had an elevation of either AST or ALT.

The mean AST and ALT levels in infants without and with aminotransferase elevations were 34.1 ± 9.5 IU/L vs. 129.0 ± 218.7 IU/L (*P *= .001) and 23.2 ± 7.9 IU/L vs. 131.4 ± 194.0 IU/L (*P *< .001), respectively. No differences were observed between infants without and with aminotransferase elevation in terms of the mean haemoglobin levels, platelet counts, neutrophil or lymphocyte differential counts, total bilirubin and C‐reactive protein levels. However, the mean white blood cell count (10 837.2 ± 4535.3/µL vs 12 565.6 ± 4935.7/µL, respectively; *P *= .010) and albumin level (4.2 ± 0.3 g/dL vs 4.3 ± 0.2 g/dL, respectively; *P *= .029) were significantly lower in infants without aminotransferase elevation than in those with aminotransferase elevation.

### 3.3 First laboratory follow‐up

**Figure 1:**
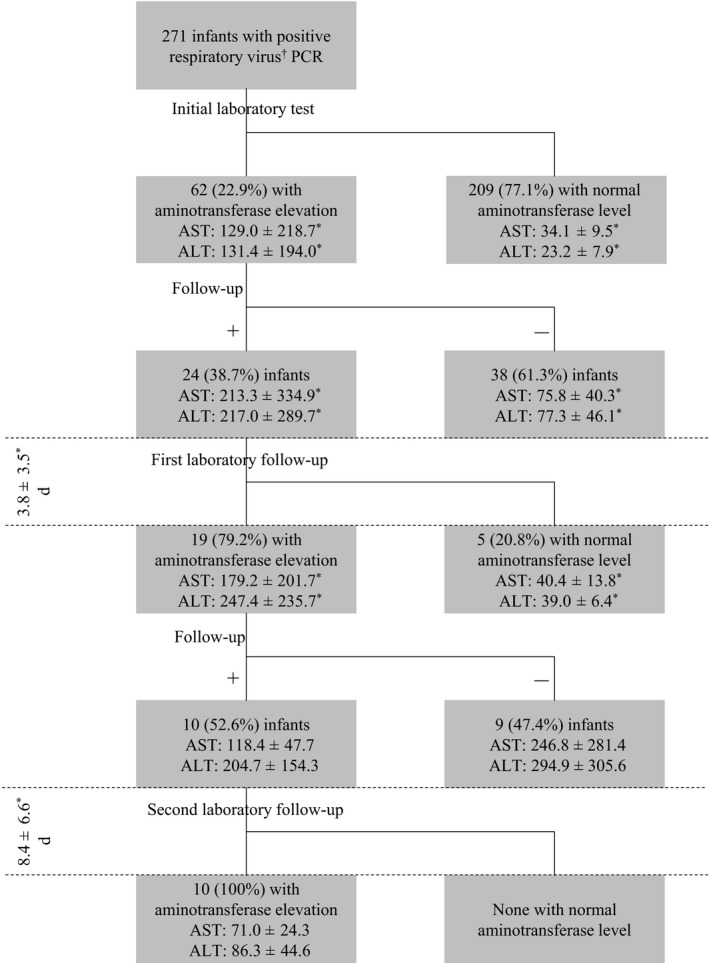
Flow and comparison of aminotransferase levels (IU/L) in febrile infants aged 90 days or less, with a positive viral PCR. Aminotransferase elevation was defined as elevation of either AST or ALT, unless otherwise specified. Comparisons were made between similar aminotransferase types at similar time points and for duration between consecutive laboratory tests using paired‐samples t test or Mann‐Whitney U test, as appropriate. Significance is represented by *P < .05. †Respiratory viral infections included in the aminotransferase testing were as follows: adenovirus, rhinovirus, enterovirus, influenza A virus, influenza B virus, parainfluenza virus 1, parainfluenza virus 2, parainfluenza virus 3, parainfluenza virus 4, respiratory syncytial virus A, respiratory syncytial virus B, bocavirus 1/2/3/4, metapneumovirus, coronavirus 229E, coronavirus NL63 and coronavirus OC43. PCR, polymerase chain reaction; AST, aspartate aminotransferase; ALT, alanine aminotransferase

The mean aminotransferase levels from the initial laboratory test in infants with (24 of 62, 38.7%) and without (38 of 62, 61.3%) first follow‐up were 213.3 ± 334.9 IU/L and 75.8 ± 40.3 IU/L, respectively, for AST (*P *= .001) and 217.0 ± 289.7 IU/L and 77.3 ± 46.1 IU/L, respectively, for ALT (*P *= .003). The 24 (38.7%) infants followed up for initial aminotransferase elevation underwent subsequent laboratory investigation within a mean interval of 3.8 ± 3.5 days from the initial laboratory test (Figure 1). Among the 24 infants, 19 (79.2%) had persistent and statistically significant aminotransferase elevation on the first laboratory follow‐up compared to the initial laboratory test. The aminotransferase levels in infants with and without aminotransferase elevation showed significant differences on the first laboratory follow‐up, with levels of 179.2 ± 201.7 IU/L and 40.4 ± 13.8 IU/L, respectively, for AST (*P *= .002) and 247.4 ± 235.7 IU/L and 39.0 ± 6.4 IU/L, respectively, for ALT (*P *= .003).

### 3.4 Second laboratory follow‐up

Among the 19 infants with elevated aminotransferase levels on the first laboratory follow‐up, 10 (52.6%) underwent a second laboratory follow‐up at a mean interval of 8.4 ± 6.6 days from the first laboratory follow‐up. The aminotransferase levels of the 10 infants who were to undergo second laboratory follow‐up (AST: 118.4 ± 47.7, ALT: 204.7 ± 154.3) were not significantly different from the 9 (47.4%) infants (AST: 246.8 ± 281.4, ALT: 294.9 ± 305.6) who did not have to undergo second laboratory follow‐up. All of the 10 infants showed persistently elevated aminotransferase levels (AST: 71.0 ± 24.3, ALT: 86.3 ± 44.6) on the second laboratory follow‐up, although the levels were lower than those at initial examination or the first follow‐up.

### 3.5 Further laboratory evaluations and other investigations

Among the 10 infants who persistently showed aminotransferase elevations, four underwent additional follow‐up investigations; these were performed once in three infants and six times in an infant with suspected Wilson's disease, which was eventually found to be absent. None of the 10 infants who required more than three follow‐ups were diagnosed with hepatobiliary disease.

Among the 62 infants with initial elevated aminotransferase levels, nine (14.5%) and seven (11.3%) underwent serologic testing for hepatotropic pathogens and ultrasonographic evaluation of the hepatobiliary system, respectively. Serologic testing for hepatotropic pathogens included tests for the following viruses: hepatitis A/B/C virus in 6/9 (66.6%) infants, herpes simplex virus in 7/9 (77.8%) infants, cytomegalovirus in 9/9 (100%) infants and Epstein‐Barr virus in 5/9 (55.6%) infants. Except for one infant who demonstrated a positive cytomegalovirus IgM test result, the serologic tests were found to be negative in all other cases. No hepatobiliary abnormalities were noted on ultrasonography.

### 3.6 Respiratory virus types and aminotransferase elevation

The percentage of infants with aminotransferase elevation for each type of RV was calculated (Table 2). The average percentage of aminotransferase elevation for all types of RVs was 13.3% for AST and 22.1% for ALT. AST elevation above the observed average (13.3%) was noted for AdV (33%), HRV (17.4%), FluA (18.8%), PIV1 (27.8%) and HBoV (25.0%). ALT elevation above the observed average (22.1%) was seen with AdV (22.2%), HRV (28.3%), PIV1 (44.4%), PIV4 (41.7%), HBoV (50.0%), CoV‐NL63 (25.0%) and CoV‐OC43 (27.8%). For AST or ALT elevation, AdV (33.3%), HRV (28.3%), PIV1 (44.4%), PIV4 (41.7%), HBoV (50.0%), CoV‐NL63 (25.0%) and CoV‐OC43 (27.8%) showed higher percentages than average for all RV types. The only virus that showed a statistically significant association with aminotransferase elevation was PIV1: *P *= .034 for ALT elevation and *P *= .038 for AST or ALT elevation.

**Table 2:** Patients with aminotransferase abnormalities by each virus

Respiratory Virus	No. (%)	AST or ALT elevation	AST elevation	ALT elevation	AST (IU/L)	ALT (IU/L)	AST to ALT ratio
AdV	9 (3.3)	**3 (33.3)**	**3 (33.3)**	**2 (22.2)**	53.0 ± 23.9	45.0 ± 40.3	1.5 ± 0.5
HRV	46 (17)	**13 (28.3)**	**8 (17.4)**	**13 (28.3)**	85.5 ± 248.7	74.6 ± 210.5	1.3 ± 0.4
HEV	35 (12.9)	3 (8.6)	1 (2.9)	2 (5.7)	36.5 ± 13.2	23.9 ± 12.9*	1.7 ± 0.6*
FluA	32 (11.8)	7 (21.9)	**6 (18.8)**	7 (21.9)	53.3 ± 42.9	46.7 ± 55.2	1.4 ± 0.5
FluB	3 (1.1)				31.0 ± 9.5	17.0 ± 4.4	1.8 ± 0.2
PIV1	18 (6.6)	**8 (44.4)** *	**5 (27.8)**	**8 (44.4)** *	78.6 ± 88.7	70.6 ± 88.1	1.2 ± 0.2*
PIV2	3 (1.1)				40.0 ± 11.5	27.7 ± 4.2	1.4 ± 0.3
PIV3	19 (7)	4 (21.1)	2 (10.5)	4 (21.1)	50.4 ± 51.1	34.0 ± 31.7	1.6 ± 0.4
PIV4	12 (4.4)	**5 (41.7)**	1 (8.3)	**5 (41.7)**	60.6 ± 71.1	62.4 ± 101.5	1.3 ± 0.4
RSV A	38 (14)	5 (13.2)	3 (7.9)	5 (13.2)	42.4 ± 23.6	34.6 ± 32.4	1.6 ± 0.9
RSV B	10 (3.7)	1 (10.0)	1 (10.0)	1 (10.0)	38.7 ± 31.7	29.9 ± 38.4	1.8 ± 0.8
HBoV	8 (3)	**4 (50.0)**	**2 (25.0)**	**4 (50.0)**	64.3 ± 43.1	55.1 ± 32.9*	1.1 ± 0.3
MPV	11 (4.1)	2 (18.2)	1 (9.1)	2 (18.2)	71.4 ± 117.0	79.1 ± 171.1	1.3 ± 0.4
CoV‐229E	1 (0.4)				28.0	22.0	1.3
CoV‐NL63	8 (3)	**2 (25.0)**	1 (12.5)	**2 (25.0)**	36.6 ± 16.5	30.4 ± 21.2	1.4 ± 0.3
CoV‐OC43	18 (6.6)	**5 (27.8)**	2 (11.1)	**5 (27.8)**	44.4 ± 26.5	46.6 ± 51.8	1.3 ± 0.5
Total	271	62 (22.9)	36 (13.3)	60 (22.1)	55.8 ± 111.7	48.0 ± 103.1	1.4 ± 0.6

Note

Values are presented as number (%) or mean ± standard deviation. Proportions above average are marked in bold.

Abbreviations: AdV, adenovirus; HRV, rhinovirus; HEV, enterovirus; FluA, influenza A virus; FluB, influenza B virus; PIV1, parainfluenza virus 1; PIV2, parainfluenza virus 2; PIV3, parainfluenza virus 3; PIV4, parainfluenza virus 4; RSV A, respiratory syncytial virus A; RSV B, respiratory syncytial virus B; HBoV, bocavirus 1/2/3/4; MPV, metapneumovirus; CoV‐229E, coronavirus 229E; CoV‐NL63, coronavirus NL63; CoV‐OC43, coronavirus OC43; AST, aspartate aminotransferase; ALT, alanine aminotransferase.

* *P *< .05.

The mean levels of AST, ALT and AST to ALT ratio for each virus type were measured. Among the 16 viruses, HBoV showed a statistically significant elevation in ALT (55.1 ± 32.9 IU/L, *P *= .011). HEV infected infants showed an elevation in the AST to ALT ratio (1.7 ± 0.6, *P *= .009).

## 4 DISCUSSION

The two major findings of this study are as follows: (a) aminotransferase elevation occurs in 22.9% of infants aged 8‐90 days with a PCR‐positive RV infection and (b) the chances of actual hepatic involvement following aminotransferase elevation in viral respiratory illnesses are uncommon to rare, as none of the infants in this study were diagnosed with a liver disease on follow‐up. Moreover, PIV1 infection was significantly associated with aminotransferase elevation.

Non‐specific hepatitis presenting as aminotransferase elevation is not uncommon in young children. Studies to understand the pathologic mechanisms underlying aminotransferase elevation in the paediatric population are limited. A study in 559 paediatric patients found that aminotransferase elevation was truly non‐specific in 7.5%.^9^ In the same study, infections were found to be the most common cause of aminotransferase elevations (57.8%), with viral respiratory infections (19.8%) being the most common aetiological factor, followed by acute gastrointestinal infections (10.5%). Rheumatologic, autoimmune and non‐alcoholic fatty liver disease also caused elevation of aminotransferases. Another study in 72 young children with isolated elevation of serum aminotransferases for 3‐36 months found that the condition was benign, and usually resolved within a year.^10^ The authors speculate that such patients could be followed conservatively, and invasive approaches including liver biopsy rarely contribute to the diagnosis.

Febrile infants younger than 90 days require close medical attention due to the possibility of SBIs and their rapid progression to life‐threatening medical conditions. In developed countries, the use of appropriate diagnostic aids and empiric antibiotics has lowered infant mortality. Wide implementation of conjugate vaccines and its administration have also favourably influenced the outcomes.^13^ Recently, efforts have been made to lower antibiotic use, promote early hospital discharge, and improve outpatient follow‐up of infants by identifying infants who appear well and have a lower likelihood of SBIs. Although the indications for RV mRT‐PCR or antigen detection in this age group are limited, these tests could be used to partly rule out invasive bacterial infections. Several studies provide substantial evidence for the use of the above tests in influenza or respiratory syncytial virus detection.^14^, ^15^, ^16^, ^17^ A recent study in febrile infants aged 0‐90 days by Nicholson et al further categorised RVs into mucosa‐restricted versus systemic types.^18^ Although higher than expected rates of coinfection with RVs and SBIs were found, the findings reinforce the mentioned evidence and widen the scope of use of RV mRT‐PCR.

Viral identification helps in the management of fever or bronchiolitis in infants aged 90 days or younger. However, further studies are required to assess the benefits of identifying viral infections. Blood tests, including the measurement of serum alanine aminotransferase, are frequently performed in febrile paediatric patients.^8^ Therefore, it is not uncommon to observe elevated serum aminotransferase levels; this is a challenging situation for the paediatrician, particularly with a mother recovering from recent labour, difficult vascular access in neonates, and prolonged admission for normalising aminotransferase levels, which could increase the possibility of healthcare‐associated infections.^19^

This study found the aminotransferase level to be elevated in 22.9% of febrile infants aged 90 days or less, with RV‐positive PCR; that is, the attending physician will encounter aminotransferase elevation in one of four infants diagnosed with a RV. Hence, it can be safely concluded that aminotransferase elevation is not an uncommon situation. To the best of our knowledge, no study has precisely reported on the proportion of infants with elevated aminotransferase in a similar group. Infections in infants aged 90 days or less are most commonly caused by viruses and occasionally by bacteria; therefore, it may be assumed that elevated aminotransferase levels are occasionally encountered in the routine workup of possible neonatal sepsis.

None of the 10 infants with aminotransferase elevation on three consecutive follow‐up tests was diagnosed with hepatobiliary disease. A patient with persistent aminotransferase elevation and suspected Wilson's disease was followed up for 8 months; however, the levels eventually normalised. Therefore, although the degree of concern varies based on clinical findings at presentation and the treating physician, the possibility of true hepatobiliary disease is extremely low in patients with accidental aminotransferase elevation.

The viruses that showed higher than average pan‐viral aminotransferase elevation included AdV, HRV, PIV1, PIV4, HBoV, CoV‐NL63 and CoV‐OC43. Among these, infection due to only PIV1 is significantly associated with aminotransferase elevation. A recent study implicated adenovirus, enterovirus and respiratory syncytial virus in the elevation of aminotransferases in paediatric patients.^20^ However, the mentioned study did not perform statistical analysis for virus types, and the age group was different, thus limiting study comparisons. An interesting finding of the present study is a significant association between PIV1 detection and ALT elevation, while none of the viruses showed significance for AST. This may be explained by the fact that 34 (94.4%) of the 36 infants with elevated AST also had elevated ALT. Thus, we speculate that ALT is more commonly elevated and maybe a better marker of non‐specific reactive aminotransferase in this population compared to AST; this finding is similar to the results of a previous study.^20^

In the present study, infants with elevated aminotransferases were approximately 10 days older than those with normal aminotransferases. The reason for this difference is unknown and cannot, therefore, be explained at this point. Surprisingly, we found no difference in the length of hospital stay based on aminotransferase elevation, as infants with elevated levels are likely to be admitted longer for follow‐up. A possible explanation is the availability of follow‐up tests in the outpatient department. Infants with elevated aminotransferases also had high white blood cell counts and serum albumin; however, the reason for its occurrence remains to be explored.

Not all infants with an initial aminotransferase elevation had subsequent follow‐ups. Only 38.7% of infants with initial elevation had the first follow‐up. However, 52.6% of infants with elevation on the first follow‐up underwent subsequent follow‐ups. The periods between initial laboratory testing and first laboratory follow‐ups and between first and second laboratory follow‐up were 3.8 ± 3.5 and 8.4 ± 6.6 days, respectively, implying that additional follow‐ups occur more infrequently. All infants with first and second follow‐up had persistent aminotransferase elevation. It is possible that the follow‐up duration was inadequate for observing the normalisation of aminotransferase levels. Interestingly, similar to the results of previous studies, none of the infants with positive viral PCR had a SBI.^3^, ^21^

This study has several limitations. First, the sample size was limited, particularly in view of the variety of viruses; the study results may, therefore, not be extrapolated to a larger population. The recognised PIV1 also demonstrated a relatively low statistical power (*α* = 0.05, *β* = 73%) in view of the sample size; there is a possibility that viruses with a true tendency for causing aminotransferase elevation were not detected due to an inadequate sample size. Second, this study is limited by the absence of negative controls. However, the inclusion of the general population with non‐specific aminotransferase elevation was not within the scope of this study. In addition, a negative mRT‐PCR does not necessarily imply that the infection is non‐viral. Therefore, the use of negative controls has its limitations. Further studies using negative controls are warranted, as more precise viral detection diagnostics become available. Nevertheless, this study has comprehensively reported the occurrence of non‐specific aminotransferase elevation in febrile infants aged 90 days or younger.

In conclusion, non‐specific hepatitis is occasionally observed with RV infection in infants aged 8‐90 days. ALT elevation is the most common abnormality, and a final diagnosis of primary hepatobiliary disease is rare. Invasive investigations may be reduced by increasing the cut‐off levels of aminotransferase in this paediatric group. Large sample studies are warranted to confirm our findings and evaluate the recommendations.
